# Creative Practices Embodied, Embedded, and Enacted in Architectural Settings: Toward an Ecological Model of Creativity

**DOI:** 10.3389/fpsyg.2015.01978

**Published:** 2016-01-06

**Authors:** Laura H. Malinin

**Affiliations:** Department of Design and Merchandising, College of Health and Human Sciences, Colorado State University, Fort CollinsCO, USA

**Keywords:** creativity, architectural design, embodied cognition, embedded cognition, enactive cognition, affordance, ecological psychology, niche construction

## Abstract

Memoires by eminently creative people often describe architectural spaces and qualities they believe instrumental for their creativity. However, places designed to encourage creativity have had mixed results, with some found to decrease creative productivity for users. This may be due, in part, to lack of suitable empirical theory or model to guide design strategies. Relationships between creative cognition and features of the physical environment remain largely uninvestigated in the scientific literature, despite general agreement among researchers that human cognition is physically and socially situated. This paper investigates what role architectural settings may play in creative processes by examining documented first person and biographical accounts of creativity with respect to three central theories of situated cognition. First, the *embodied thesis* argues that cognition encompasses both the mind and the body. Second, the *embedded thesis* maintains that people exploit features of the physical and social environment to increase their cognitive capabilities. Third, the *enaction thesis* describes cognition as dependent upon a person’s interactions with the world. Common themes inform three propositions, illustrated in a new theoretical framework describing relationships between people and their architectural settings with respect to different cognitive processes of creativity. The framework is intended as a starting point toward an ecological model of creativity, which may be used to guide future creative process research and architectural design strategies to support user creative productivity.

## Introduction

Stories abound about how creative people feel their physical environments become part of their creative process. Kipling (1937) described in detail the office where he wrote and espoused the importance of his “working tools,” including obsidian black ink and a camel hair brush, as the “magic” behind his creativity. Marcel Proust wrote from his childhood bed at the Haussmann Boulevard residence, in a dimly lit room where he lined the walls and ceiling with cork providing protection from dust that triggered allergies and social intrusions that might distract him from his work (Fuss, 2004). Immanuel Kant habitually gazed at the Löbenicht church steeple from the window of his home at 87–88 Prinzessinstraße and, feeling so strongly its importance to his creative process, insisted his neighbor’s tree be cut down when it grew to obscure his view (Wasianski, 1902). Jonas Salk attributed his breakthrough on the polio vaccine to time spent at a 13th century monastery in Italy. He later tasked the architect Louis Kahn with capturing its aesthetic qualities through the iconic design of the Salk Institute in La Jolla, CA, USA — hoping to similarly inspire creativity among the scientists working there (Leslie, 2008, 2010). These stories suggest that people’s creative processes may be intrinsically linked with the settings in which they work as a form of physically situated cognition, however, the potential role of the physical environment in creative processes has received little attention in the empirical literature (Drake, 2003; Dul et al., 2011). Although architects (like Kahn) have designed places to foster creativity, they do so without an appropriate theory to inform design strategies. The aim of this paper is first to inform scholarly discourse around the topic of creative cognition as embodied, embedded, and enacted in architectural settings and second to provide a theoretical framework illustrating relationships between people and their physical environments during creativity, guiding future research, and architectural design strategies supporting user creative productivity.

This paper examines first person and biographical accounts of creative practitioners that describe their creative processes, including what they do and how they work in architectural settings when solving ill-defined problems. *Creative practitioner^1^* is a term used to include extraordinarily creative people as well as professionals earning a living through creative work, for example, artists, writers, composers, choreographers, architects, scientists, and anyone who has developed sufficient domain expertise to be compensated for developing ideas or products. Domain expertise is necessary to be creative within a field (Csikszentmihalyi, 1996; Runco, 2007) and the ability to earn a living addresses the varying years of practice required by different disciplines. Creativity involves stages from problem identification through ideation and implementation of a product (artifact, theory, technique, process, etc.) that is original and has value or purpose for a segment of society. *Creative processes* examined in this paper include those involved in ideation stages, defined here as generating, elaborating, and incubating. Finally, *architectural settings* are designed environments defined by features and qualities relevant to building design professionals, such as: (a) building sites and their connectivity to regional amenities (e.g., walking or bicycle paths, streets, bus or train stops, etc.); (b) building structures including materials, spatial layout, and orientation to views or other site amenities; and (c) rooms and their finishes, furnishings, equipment (e.g., lighting), and shared or personal items (e.g., tools, materials, and decorative objects).

The argument presented here is theoretically grounded in empirical knowledge from cognitive science, ecological psychology, and the creativity and design literatures (including psychological and neurobiological studies). It is organized as follows: first, a review of creativity research approaches highlights the gap in the literature as it concerns the physical context of creativity. Next, theories of embodied, embedded, and enacted cognition (the 3E’s) are used to examine relationships between creative cognition and architectural settings. The 3E’s serve to organize first person and biographical accounts of creativity around common physically situated processes. This organization informs three propositions about person–environment relationships during creativity. The propositions are illustrated in a new conceptual framework, integrating and extending prior theoretical work in enactive cognition by Varela et al. (1991) and ecological psychology by Gibson (1977). The framework describes the dynamic relationship between people and features of their architectural settings during situated processes of creativity, providing foundational work for an ecological model aimed at better understanding and predicting creative behaviors in designed environments.

## Considering the Physical Context of Creativity

Scientists acknowledge that creativity is a complex and multifaceted phenomenon that cannot be fully understood from the perspective of a singular approach or domain of study (Runco, 2007; Sawyer, 2012), yet the physical context of creativity has received relatively little attention in the literature (Hunter et al., 2007; Dul et al., 2011). In fact, much research conducted over the past century has focused only on certain aspects of creativity (Fryer, 2012), organized by Rhodes (1961) as the *Four Ps*: Person, Product, Process, and Press (environments supporting creativity). Within the press research strand there have been some efforts to understand how creative performance results from interactions between different dimensions of creativity, including social (but not physical) environments^2^. Process research strands largely focus on purely mental operations, with consideration for the socially situated nature of certain creativity stages reflected in some recent models^3^. Whether creative processes are also physically situated remains largely uninvestigated, with the notable exception being Csikszentmihalyi’s (1990, 1996) flow theory, describing a single creative process. From the architectural design perspective, there have been a few attempts to understand impacts of workplace designs on user creative productivity, however, studies are often limited to participant perceptions regarding social interactions or aesthetics. There is little evidence of meaningful integration between creative process and physical press research strands.

### Architectural Design Strategies Promoting Creativity

Social behaviors are frequently promoted in modern architectural designs intended to increase creativity. For example, in his design for the Salk Institute, Kahn separated scientists’ offices (inspired by monastic cells at Assisi) from their laboratories, providing courtyard gardens between to host impromptu conversations he envisioned occurring when people walked between their workspaces (Kahn, 2003, pp. 71, 132–134, 142–145). Office buildings incorporate strategies to increase communication and collaboration by encouraging social density in ‘attractor’ spaces such as workrooms, atriums, and cafés (Fayard and Weeks, 2007; Yaneva, 2010; Sailer, 2011) and eschewing private offices in lieu of open office designs (Ekvall and Tångeberg-Andersson, 1986; Vithayathawornwong et al., 2003; McCoy, 2005). Research examining effects of these strategies is minimal and results contradictory (McCoy, 2005; Fayard and Weeks, 2007). Some studies find better information and idea exchange in private offices than multi-purpose rooms, cafés, meeting rooms (Grajewski, 1993), or open offices (Hatch, 1987; Vithayathawornwong et al., 2003) and others discover increased quantity and frequency of social interaction in open offices, but higher quality of communication (Ekvall and Tångeberg-Andersson, 1986) and greater creativity (Sailer, 2011) in private offices. There is no clear evidence of how spatial configurations might support creativity. Further, studies do not consider the full range of creative behaviors involved in different stages of creativity, focusing only on communication and social interactions as predictors of creative productivity.

Aesthetic qualities people believe inspire their creativity are a frequent subject in first person accounts, and studies show those environments people perceive as inspirational generally do increase their creative productivity (McCoy and Evans, 2002; Dul and Ceylan, 2011; Dul et al., 2011). However, identification of specific architectural features or attributes remains elusive. Scientists have examined people’s preferences for different design features in meeting rooms (Ceylan et al., 2008; de Korte et al., 2011) libraries, offices, living rooms, hallways, dining facilities, sports facilities, and retail stores (McCoy and Evans, 2002). Findings suggest people prefer rooms with natural lighting and views of nature (McCoy and Evans, 2002; Ceylan et al., 2008), but color and material choices are unclear; people preferred warm colors and materials high in visual complexity in one study (McCoy and Evans, 2002) but cool colors and low visual complexity in another (Ceylan et al., 2008). Hypothesizing that spatial arousal effects impact creative ideation, de Korte et al. (2011) found although red rooms are more arousing than blue and green rooms (as measured by heart rate variability), room color did not significantly impact ideation fluency. Mehta et al. (2012) find ideational fluency and originality improves in conditions with moderate background noise (such as found in a café), however, effects are not likely due to spatial arousal as first hypothesized, but processing disfluency (low level distraction) which increases abstraction, reduces confirmation bias, and consequently improves ideation. Anecdotes describe different types of places as creativity unfolds; yet impacts of spatial qualities on behavior and cognitive processes during different stages of creativity (from problem finding through product implementation) remain largely uninvestigated.

### Process Models and Their Limitations for Informing Architectural Designs

Scientific understanding of creative processes has largely been informed by studying what eminently creative people do (or say they do)^4^ (Sternberg, 1999). Wallas (1926) developed one of the earliest and most enduring stage models from first-person accounts of creativity — a speech by German physicist Hermann von Helmholtz (pp. 79–80) and a book chapter written by the French mathematician Jules Henri Poincaré (p. 75). His model describes creativity as conscious (explicit) and subconscious (intuitive) mental processes involving stages of: (1) preparation, where knowledge is acquired; (2) incubation, a period of rest when knowledge is subconsciously restructured; (3) illumination, a moment of insight; and (4) verification, when an idea is evaluated and possibly applied. The Wallas model continues to be extensively referenced^5^ in the creativity literature despite criticisms it (a) neglects to identify all sub-processes of creativity and (b) does not adequately explain relationships between stages including how people sequence between them (Lubart, 2001; Fryer, 2012). This paper argues another limitation is it reduces creativity to mental operations, giving little consideration for physically situated processes.

Many researchers have developed their own process models attempting to address limitations of the Wallas model, including those cited in **Table 1.** Some identify additional sub-processes of creativity by dividing Wallas’s preparation (Osborn, 1953; Sternberg et al., 2002) or verification stages (Feldman et al., 1994; Csikszentmihalyi, 1996; Sternberg et al., 2002). Others propose entirely new stages, such as Evans and Russell’s (1989) *frustration* stage or Sternberg’s (2006) *redefine problems* (first) and *sell idea* (last) stages. Many models reflect a shift in thinking about creativity from purely individual to a socio-cultural process, incorporating social activities such as brainstorming (Osborn, 1953) for ideation and feedback from critique or use during implementation (Van Gundy, 1987; Mumford et al., 1991; Feldman et al., 1994; Isaksen et al., 2000). This social aspect of creativity is often reflected in modern workplace designs; however, *when and how during the creative process social interactions improve (or inhibit) creativity remains unclear*. For example, studies find brainstorming groups are less effective at generating ideas than the same number of people working alone (Diehl and Stroebe, 1987; Diehl and Stroebe, 1991; Mullen et al., 1991; Kohn and Smith, 2011), however, many people attribute social interaction to breakthrough on a creative problem (Johnson, 2010). Modern workplace designs are largely based in trends emphasizing ‘attractor’ spaces to provoke social interactions, with little understanding about how social interactions engender, support, or inhibit different creative processes, or sequences of processes — nor do they measure impacts these spaces have on innovation and organizational performance” (Waber et al., 2014).

**Table 1:** Creative stage models compared.

	Modes of creative thinking				
	
Model Author(s)	Problem-finding/Problem-framing	Ideation			Implementation/Feedback from use
			
		Generating	Incubating	Elaborating	
Wallas, 1926	Preparation		Incubation, Illumination	Verification	
Rossman, 1931	Observation, Analysis, Survey	Formulation, Critique, Invention		Experimentation, Selection, Perfection	
Osborn, 1953	Orientation, Preparation, Analysis	Hypothesis	Incubation	Synthesis, Verification	
Gordon, 1961	Groundwork	Immersion, Divergent Exploration		Selection, Articulation, Transformation	Implementation
Bransford and Stein, 1984	Identify Problem, Define Goals	Explore Approaches		Act on Plan	Look at Effects
Van Gundy, 1987	Objective Finding, Fact Finding, Problem Finding	Idea Finding		Solution Finding	Acceptance Finding
Barron, 1988	Conception		Gestation, Pasturation	Bringing Up Baby	
Evans and Russell, 1989	Preparation, Frustration		Incubation, Insight	Evaluation, Elaboration	
Csikszentmihalyi, 1990, 1996		Flow			
Mumford et al., 1991	Problem Construction, Knowledge Acquisition, Concept Selection	Novel Combination, Ideation		Evaluation	Implementation and Feedback
Finke et al., 1992		Generative		Exploratory	
Feldman et al., 1994	Internalize Domain	Generate Novelty		Externalize Ideas	Submit to Field, Evaluate, Disseminate
Isaksen et al., 2000	Frame Problems, Explore Data, Construct Opportunities	Generate Ideas		Develop Solutions	Build Acceptance


Process models are explanatory, describing sequential stages of creativity; although they often inform creativity training approaches, they have not had much predictive power (Runco, 2007; Sawyer, 2012). The creative process is understood to be iterative, suggesting people move through stages multiple times, possibly out of sequence (Armbruster, 1989; Csikszentmihalyi, 1996), and as they deem appropriate (Lubart, 2001). There are few models that consider relationships between stages. Evans and Russell (1989) suggest during the preparation stage the mind eventually reaches a limit to the amount of information it can absorb, leading to a frustration stage, which then incites an incubation stage. Finke’s (1997) Genoplore model describes complementary generative and exploratory processes during ideation. People generate initial ideas, which he describes as incomplete plans, and test these through exploratory actions. Outcomes of exploration are used to develop the idea, generating new exploratory actions, and so forth, until the creative product emerges from the process. Csikszentmihalyi’s (1990, 1996) flow theory describes complementary processes of thinking and acting when people feel immersed in a creative experience and at their most productive. During flow, people maintain undivided attention to the task at hand, externalize a creative idea through making, perceive immediate feedback from their exploratory actions or strategies, and have a sense of personal enjoyment while engaged in the experience (Csikszentmihalyi, 1996, pp 110–113). Flow is described as a mental state of creativity, but is engendered through physically situated activities and sustained by specific environmental conditions. Flow requires significant mental effort; people rely on familiar tools and materials to sustain attention and prefer comfortable settings to help them focus (Csikszentmihalyi, 1996, p. 120). As a mode of physically situated cognition, flow may begin to provide insight into why creative people attribute importance to particular settings or features of their physical environment. Flow theory does not account for other stages (or modes of situated cognition) that occur throughout the creative process.

Popularity of the Wallas model persists, researchers suggest, because it (a) describes what eminently creative people have written about their creative process, (b) it resonates with what people feel they do when they are creative, and, (c) although researchers have sought to address its limitations, they have yet to provide a better model (Armbruster, 1989; Fryer, 2012). Because it was developed over a century ago, it does not reflect new knowledge from brain sciences, including how people leverage social and physical resources in their environments to improve cognition. As a starting point toward better understanding the physically situated processes of creativity, **Table 1** organizes commonly cited stage models, and Csikszentmihalyi’s physically situated process of creative flow, around common *modes of creative thinking* they describe. This organization guides analysis of first person and autobiographical accounts of creativity for evidence of physically situated processes. Modes are organized as follows. *Problem-finding* categorizes all stages prior to novel idea ideation, including problem definition/framing and knowledge acquisition (e.g., the Wallas preparation stage). *Ideation* includes *generating* stages describing processes for coming up with new ideas, *incubating* stages involving subconscious processes when people are not explicitly working on a problem, and *elaborating*, characterized by stages of verification, articulation, selection, and refinement. *Implementation* involves stages when a creative idea is tested and evaluated in a socio-cultural context. This paper focuses on the three modes of ideation.

The following section considers how creativity may be physically situated; documented accounts by creative practitioners are organized by mode of creative ideation they describe and examined with respect to the situated cognition theories of embodied, embedded, and enactive cognition. The intention behind this effort is to better understand if/how features and qualities of the physical environment constrain and/or enable creative ideation.

## Physically Situating Creativity with the 3E’s

**FIGURE 1:**
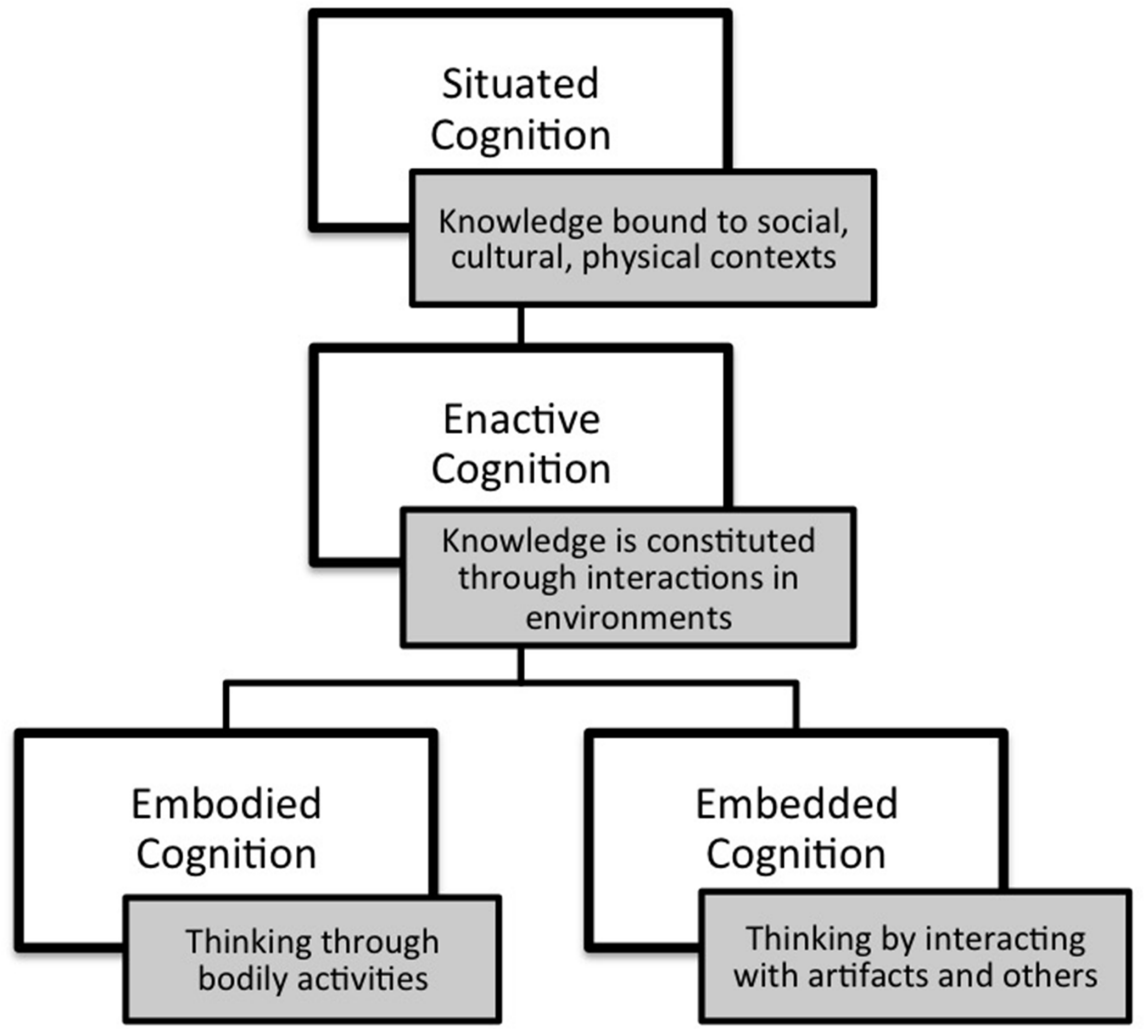
**Situated cognition and the three E’s**.

Environmental structure is now understood to be critical to human cognition^6^ (Thagard, 2005; Leidlmair, 2009; Robbins and Aydede, 2009) and situated cognition theory describes knowledge as “inextricably situated in the physical and social context of its acquisition and use” (Brown, 2001, p. 65). Three central ideas^7^ in situated cognition (the 3E’s) consider how cognition is *physically* situated: (1) *the embodied thesis* – that cognition encompasses both the mind and the body (Varela et al., 1991; Lakoff and Johnson, 1999; Gallagher, 2005); (2) *the embedded thesis* – that people exploit features of the physical and social environment to increase cognitive capabilities; (Kirsh and Maglio, 1994; Clark, 2008a), and (3) *the enactive thesis* — that cognition is constituted through a person’s actions in the world. Enactive cognition is generally treated as a theory separate from embodied and embedded cognition, however, Ward and Stapleton (2012) argue if cognition is enactive it is also embodied and embedded. Enactive cognition serves here as an overarching theory, focusing attention on the *importance of action* in ways people implicitly understand how settings provide resources for thinking-in-action. **Figure 1** defines and describes relationships between the 3E’s to guide analysis of first person and biographical accounts for evidence of physically situated cognition. In the next section the 3E’s are discussed separately, focusing attention on common themes describing person–environment relationships during different modes of creativity. Later in the paper, findings are summarized in a table which associates 3E theories with modes of creative cognition, illustrating how embodied, embedded, and enactive processes are integrated within the modes.

### Embodied Experiences in, Between, and with Architectural Settings

The embodied thesis maintains cognition depends upon physical characteristics of the body (Wilson, 2002); its sensory and motor capabilities shape the mind (Robbins and Aydede, 2009). The thesis has philosophical roots in existential phenomenology works of Edmund Husserl, Maurice Merleau-Ponty, and Martin Heidegger who, among others, believed the body is central to perception and experience (Varela et al., 1991; Gallagher, 2009; Pallasmaa, 2010). With their book *The Embodied Mind*, Varela et al. (1991) proposed a phenomenological study of cognition considering physical characteristics and abilities of the body in determining how and what sensorimotor knowledge people are able to construct through interactions in their environments. Influences of phenomenology and embodiment are found today in architectural designs by Zumthor (Mallgrave, 2013), Holl, and Pallasmaa (Holl et al., 2006), with Pallasmaa largely responsible for bringing awareness of embodied cognition to architectural design (Mallgrave, 2011) by advocating for multi-sensory environments engaging hearing, smell, and touch as antidote to the visual bias in architecture, which he believes yields “impoverished environments” causing feelings of detachment and alienation in users (Pallasmaa, 2005).

**FIGURE 2:**
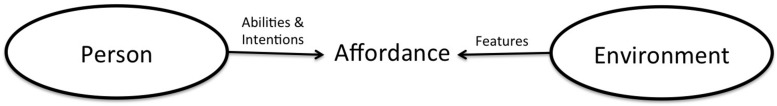
**Affordance according to Gibson (1977), is a transactional relationship between animal (e.g., person) and environment**.

Another historical influence for the embodiment thesis is Gibson’s (1977) work in ecological psychology, including his *theory of affordances*. Gibson believed people understand the world in terms of functional relevance and possibilities for action (affordances). Although Gibson (1976, p. 413) proposed that his ecological thesis could provide a much-needed foundation for architecture, affordance theory has been more influential in product design.^8^ Gibson defines affordance as a *relationship* between person and environment, dependent upon the person’s intentions and physical abilities with respect to *action opportunities* provided by features of the environment (**Figure 2**). From his perspective, knowledge is constructed through goal-directed exploratory actions. The “bottom-up” approach to cognition he describes is reflected in Brooks’s (1991a,b) seminal artificial intelligence research and Clark’s (2001) concept of “intelligence without [mental] representation,” arguing minds are not for *thinking* but for *doing*. Some in the architectural design community champion affordance theory to better predict behavioral outcomes of designed spaces (Maier et al., 2009; Lang and Moleski, 2010), however, there is little evidence of its effectiveness *in practice*.

A key difference between the philosophical and ecological approaches to embodiment in architectural design is the concept of user agency. The philosophical perspective is more concerned with how the body *constrains cognition* (Borghi and Cimatti, 2010), emphasizing ways spatial features and attributes affect user (subjective) experiences. In architecture the user is viewed as a passive recipient of design interventions. The ecological perspective considers the role the body plays in *constituting cognition* (Reed, 1996) suggesting people actively exploit features and attributes of architectural settings as part of their cognitive system, taking ownership of their experiences in their settings. The ecological perspective is most clearly evident in creativity narratives, including those of Kipling, Kant, and Proust mentioned in the introduction to this paper. Two overarching themes of the ecological approach emerge from personal accounts of creativity: first, people use artifacts in their environment (e.g., tools and materials) as transparent equipment shaping perceptions during intuitive ideation, and second, people personalize their settings to help initiate and sustain creative flow by incorporating them into ritual and sense of creative self.

#### Thinking-in-Action: *Seeing Through* Tools and Materials

People feel tools and materials used during creative ideation become an extension of themselves, serving to organize creative experiences (Sennett, 2008; Pallasmaa, 2010). Accounts by creative practitioners suggest artifacts in their environment are embodied when they (a) are customary and familiar, (b) facilitate thinking-in-action (such as through writing, drawing, and model making), and (c) deepen immersion in the creative process by enabling immediate feedback from exploratory actions, shaping experiences in a creative situation.

*Personal narratives describe strong feelings for tools and materials^9^* with some seeming almost superstitious about roles they play in creative performance. Kipling (1937) describes how his creative “Daemon” responds to particular writing instruments and materials, expressing distress when the nib of a favored pen failed him during “an evil hour.”

And with what tools did I work in my own mold-loft?…I used a slim, octagonal-sided, agate penholder with a Waverley nib. It was a gift, and when in an evil hour it snapped I was much disturbed…For my ink I demanded the blackest…All blue-blacks’ were an abomination to my Daemon…My writing-blocks were built for me to an unchanged pattern of large, off-white, blue sheets…With a lead pencil I ceased to express— probably because I had to use a pencil in reporting. — Kipling (1937)

We surmise from his writing that Kipling (1937) has come to favor certain tools and materials; he uses them regularly (they are customary), and expertly (they are familiar) so they do not distract from his writing. A favorite pen has a tight relationship with the way he thinks creatively; he *sees through* the tool to the creative situation. When such a tool fails to perform as expected, his creative process is negatively impacted, the tool no longer transparently part of his thinking. Instead it becomes a distraction, a source of distress. *Creative practitioners describe intuitively thinking-in-action when tools and materials are fluidly incorporated into their idea-generating processes* through activities like sketching, drawing, writing, and model making. Externalizing an idea allows people to perceive “feedback” from the situation, identifying unanticipated opportunities in a creative situation and initiating new actions in response to them (Schön, 1983, pp 163–164). Aalto (1997, p. 108) describes his process of intuitively sketching to reconcile the complex and contradictory requirements of an architectural design:

“I forget the whole maze of problems for a while, as soon as the feel of the assignment and the innumerable demands it involves have sunk into my subconscious. I then move on to a method of working that is very much like abstract art. I simply draw by instinct, not architectural synthesis, but what are sometimes quite childlike compositions, and in this way, on an abstract basis, the main idea generally takes shape, a kind of universal substance that helps me to bring the numerous contradictory components into harmony.”

Sketching is used to (1) “handle different levels of abstraction simultaneously,” (2) “enable identification and recall of relevant knowledge,” (3) “assist problem structuring through solution attempts,” and (4) “promote the recognition of emergent features and properties” of the design idea (Cross, 2006, p. 37). Studies have shown the tight relationship between thinking and acting with tools — demonstrating activation of motor processes in the brain when people think about using tools, say words associated with tool use, or watch someone else use a tool during experimental tasks (Pulvermüller et al., 2005; Mahon and Caramazza, 2008). Intuitive process of thinking-in-action is described in Csikszentmihalyi’s (1990, 1996) flow theory, with understanding gained through unselfconscious participation and direct experience in a creative situation. The improvisational jazz performer anticipating each new note as he hears the last one played (Schön, 1983, pp 55–56), the painter responding to the texture of the paint and the colors of pigment on a canvas as she positions the brush to make the next stroke (Csikszentmihalyi, 1996, p. 208), and the scientist working through the structure of DNA by manipulating and reconfiguring a physical model of machined parts (Watson, 1968, pp 193–197) are examples of flow. In these cases there is fluid intertwining of action and perception, and understanding comes from first-hand experience in a physical context.

*The ability to immediately perceive feedback from exploratory actions deepens feelings of immersion in the creative situation*, blurring the boundary between creator and creation. Berger (2005, p. 3) describes how he becomes more and more immersed in the creative process through drawing until he feels he and the product of his creation have merged.

Each confirmation or denial brings you closer to the object, until finally you are, as it were, inside it: the contours you have drawn no longer marking the edge of what you have seen, but the edge of what you have become.

Each new action is a response to the current set of circumstances; ensuing immersion in the process characterized as activity involving “continual reciprocal causation” (Clark, 2008b, p. 24) when Berger (2005) is simultaneously affecting his situation and being affected by it. Clark (2008b, p. 25) describes a famous exchange between physicist Richard Feynman and historian Charles Weiner to illustrate this principle. Feynman argues with Weiner that a paper he wrote is not a record of his thinking, but actually *is* his thinking. Feynman’s use of pen and paper is “responsible for the shape of the flow of thoughts and ideas.” Jung (1952, p. 230) eloquently describes a similar relationship between Goethe and his writing as follows: “*The work in process becomes the poet’s fate and determines his psychic development. It is not Goethe who creates* Faust*, but* Faust *which creates Goethe*.”. In these cases sense of creative self extends beyond the body to materials of creative ideation.

***Theme 1: Tools and materials are ‘transparent equipment’ when people* see through them *to the task at hand, extending sense of the body during intuitive immersion in ideation activities.***

Widely accepted is that that body schema, somatosensory representation of the body, changes with tool use (Cardinali et al., 2009). Extended capabilities afforded by a tool are reflected in neural networks in the brain as corporeal awareness of the body changes (Maravita and Iriki, 2004). Body schema is highly plastic, rapidly adapting to new tool use (Carlson et al., 2010) and can persist for years, such as the case with phantom limb syndrome or prosthetic device usage (Mayer et al., 2008). Gallagher (2005) distinguishes between body image (a conscious sense of ownership) and body schema (an intuitive sense of sensorimotor capabilities involved in interacting with the environment). When a tool becomes part of the body schema, it acts as *transparent equipment* —the user *sees through* the tool to the task at hand (Clark, 2008b, p. 10) and when this tool is misplaced or fails to perform (such as when Kipling’s Waverly nib snapped,) a person may feel temporarily handicapped (e.g., “much disturbed”) over perceived loss of creative capabilities. (Is a painter still a painter if you take away his brush?)

#### Sense-Giving Spaces for Initiating and Sustaining Creative Flow

Creative people are often as particular about their working spaces as they are with their tools. They personalize workspaces, populating them with meaningful objects or orienting furniture to favored views, to help them get into a creative mindset, incorporating environmental features and artifacts into rituals and sense of creative self. The role of inspirational objects in ritualistic creative behaviors is the subject of many narratives (Fig, 2009). Kipling (1937) wrote an entire chapter devoted to the significance of his “working tools.” His tools included meaningful objects from travels kept on his desk he felt were instrumental to his creativity. He explains how these items are essential for influencing his creative thoughts.

… I always kept certain gadgets on my work-table, which was ten feet long from North to South and badly congested. One was a long, lacquer, canoe-shaped pen-tray full of brushes and dead ‘fountains’; a wooden box held clips and bands; another, a tin one, pins; yet another, a bottle-slider, kept all manner of unneeded essentials from emery-paper to small screwdrivers; a paper-weight, said to have been Warren Hastings’ a tiny, weighted fur-seal and a leather crocodile sat on some of the papers; an inky foot-rule and a Father of Penwipers which a much-loved housemaid of ours presented yearly, made up the main-guard of these little fetishes…. Left and right of the table were two big globes, on one of which a great airman had once outlined in white paint those air-routes to the East and Australia which were well in use before my death — Kipling (1937)

Creative practitioners often develop routines to begin creative processes, such as cleaning up work surfaces or setting out favorite tools or meaningful artifacts (Csikszentmihalyi, 1996, pp 351–358; Fig, 2009). When productivity lags they change routines, alter features of their workspace, or move to a new setting (Fig, 2009). Although the white, empty art studio may be a figural representation of a creative space as a ‘blank slate’ where anything might happen, in reality the places artists work are often sensory-rich and full of tools, materials, and other inspirational objects (Fig, 2009). Aspects of architectural settings — inspirational objects, room configurations, and views — may play an important role as stimulus to beginning creative ideation. The sustained and focused attention required for flow takes significant effort (Csikszentmihalyi, 1990, pp 30–33, 54); people often feel that they need to overcome psychological barriers to begin the process (Csikszentmihalyi, 1996, pp 344–346).

Environmental features become part of the creative practitioner’s cognitive system during ideation when they function without distracting attention from the creative task. In this respect, qualities of the environment may fall under Murray’s (1938) definition of *alpha press*, when people perceive more than they attend to (Noë, 2004). Through habitual incorporation of specific environmental features and qualities into creative processes, people feel they become an integral part of their creative self. The tower view from his writing table seems to have served this purpose for Kant, as he purportedly became distraught when his neighbor’s tree obscured his view, insisting it be cut down (Wasianski, 1902). For Proust, who suffered from allergies and asthma, his cork-lined room initially provided ideal conditions for health and privacy, but eventually the womb-like space devoid of sensory stimulation became an essential part of his twelve-year effort writing about time and space (Fuss, 2004). In cases like these, spatial features and qualities of settings appear integral to creative processes, influencing ideation and sense of self.

**Theme 2: People incorporate features and sensory attributes of settings into ritualistic behaviors to psychologically prepare for creative efforts, integrating them into concept of creative self over time.**

The philosophical perspective of embodied cognition is supported when settings function as transparent equipment, part of a body’s sense-making process during creativity. Whether ambient sounds, motions, or inspirational views are truly embodied, to the extent they are incorporated into the body schema, cannot be determined from anecdotal description — although people feel they are. In personalizing their settings, people create their *cognitive niche* for creativity. Through “cognitive niche construction…[people] build physical structures that transform problem spaces in ways that aid thinking and reasoning” (Clark, 2008b, p. 62). Features and attributes of workplaces become resources in the cognitive niche improving creative abilities, whether or not they are incorporated into body schema. The concept of niche construction, however, fits more closely with the embedded thesis when people manipulate features and attributes of the environment in order to extend creative capabilities.

### Architectural Settings as Scaffolding for Embedded Cognition

Embodied and embedded cognition often go hand-in-hand and are sometimes referred to collectively as embodied, embedded cognition (Clark, 2008a). Where embodied cognition considers how people use their bodies to help them think, embedded cognition theory considers how people use features of their environment to improve their cognitive abilities (Robbins and Aydede, 2009), including how they off-load cognitive work to their environments (Clark, 2001). Clark (2001, p. 46) refers to this as the “007 Principle” meaning “know only as much as you need to know to get the job done”. People will not store or process information they can easily off-load to the environment, a process of *cognitive bootstrapping* (Clark, 2008b). Stories describe how people exploit aspects of their environments as *things to think with*, helping them better understand, evaluate, and elaborate on ideas. They employ strategies of (a) *seeing with* different tools and materials to perceive hidden affordances in a situation through abstraction, (b) *seeing as* objects and qualities of their settings to redefine or reframe a problem or idea, and (c) seeking out new environments with different resources to feed their creativity.

#### Things to Think With: *Seeing With* and *Seeing As*

People use resources in their environments as cognitive strategies to simplify the complexity of creative problems through abstraction with different materials, or externalizing ideas in different ways, by *seeing with* a variety of tools and materials or to identify new opportunities in a situation or by *seeing as* another situation (e.g., using precedents or analogy), helping to reframe an idea or problem. Reflecting on discovery of the structure of DNA with Watson (1968), Crick (1990) describes two essential factors for creative success — the ability to find (or define) an interesting problem and perseverance and skills required to consider it from multiple perspectives, using all available resources.

The major credit I think Jim and I deserve… is for selecting the right problem and sticking to it…. Both of us had decided, quite independently of each other, that the central problem in molecular biology was the chemical structure of the gene…. We could not see what the answer was, but we considered it so important that we were determined to think about it long and hard, from any relevant point of view. — Crick (1990, pp 74–75)

In their respective autobiographies, Watson (1968) and Crick (1990) describe myriad of different resources and perspectives they used in their work, including diagramming, writing, conversations with other scientists, and, most importantly, physical model building. It was through iterative manipulation of three-dimensional materials that they finally discovered the structure of DNA. Just as tools organize the creative imagination, so too are materials and methods used to simplify, externalize, and evaluate a creative idea when people ‘see with’ them to uncover previously unperceived opportunities or constraints in a situation. Diagrams are a visual method of abstracting and compressing information (Garcia, 2010, p. 18) used to understand or analyze relationships (e.g., temporal, spatial, or organizational) or generate form through conceptual representation (Allen and Agrest, 2009, pp 41–69; Eisenman, 2010). Diagrams generally focus more on describing structural relationships than meaning making (Allen and Agrest, 2009, p. 50), yet their abstracted nature may facilitate deeper understanding about a creative problem or idea through analogy and conceptual combinations (Kazmierczak, 2003). In architecture and engineering different types of drawings (plans, sections, elevations, perspectives) isolate select spatial relationships for examination (Allen and Agrest, 2009, pp 3–40; Evans, 2000). Models are used in many disciplines, including mathematics and science, to help people better understand three-dimensional relationships. Model making was instrumental in helping Watson (1968, pp 193–197) and Crick (1990) work through the structure of DNA as they manipulated and reconfigured various machined parts. Diagrams, drawings, and models facilitate *epistemic actions*, defined by Kirsh and Maglio (1994, p. 513) as “actions performed to uncover information that is hidden or hard to compute mentally.” When problems are particularly challenging, epistemic actions aid in the understanding of a problem, with incremental insights gleaned through feedback from environmental conditions.

People employ a method of *seeing-as* to focus on particular aspects of the creative situation, filtering out any detail that may obscure or confuse their ability to perceive affordances in the situation by seeing one case as another previously experienced case (i.e., precedents), or by comparing experiences in one situation with their experiences in a different, unrelated situation (i.e., analogy). Analogy is a frequently described cognitive process of creativity that involves transferring the cognitive structure from one context where it is well established to a new context where it had never been used. Dunbar (1995) describes three types of analogical thinking: selective comparison, local analogy, and regional analogy. These processes differ in the domain distance between the two contexts. Selective comparison uses different cognitive structures from within the same domain. Local analogy involves application of a cognitive structure from one domain to a related domain. Regional analogy involves transferring the cognitive structure between completely dissimilar domains.

Many acts of extraordinary creativity involve regional analogy and people often describe using aspects of their physical environments to help them make conceptual leaps. Le Corbusier’s design for the roof of Notre Dame du Haut was inspired by a crab shell he had picked up on the beach and noticed laying on his drawing board next to the building sketches (Groat and Wang, 2002, p. 102). The architect John Utzon used experiences in his environment to help him think about his design for the Sydney Opera House (Peltason and Ong-Yan, 2010, pp 91–97). He watched large ships being built with ribs in the shipyard outside his office building. He considered how the fruit of an orange is organized in sections. He imagined how space inside a building was like music. All of this information acquired from the environment changed the way he approached the design for the iconic building and influenced its form, organization, and structure. Philo Farnsworth was plowing a field when he came up with the idea to project moving images line-by-line — which led to the invention of the television (Thomas, 2004). George de Mestral found inspiration for Velcro as he picked burrs off of his dog after a walk in the woods (Hargroves and Smith, 2006). Creative practitioners seem particularly skillful at exploiting environmental resources to help them consider creative problems and ideas from many different perspectives, often leading to leaps in insight as they solve complex problems.

**Theme 3: People use methods of *seeing with* tools and materials and *seeing as* objects and features of their environment to understand complex problems and creative situations in new ways.**

Creative practitioners shape their own creative situations by acting in and on their environments, but the situations they create, in turn, influence their experiences and affordances they are able to perceive. Some stories suggest they are attuned to search their environment for potentially relevant information, even when not explicitly working on a problem — for example, Farnsworth’s ability to perceive affordances in the way he plowed a field for his pioneering work in television or de Mestral’s idea for Velcro from the hooked structure of a plant he pulled off his pet.

#### Serendipity Favors the Embedded Mind

People are active agents, explorers of their environment who habitually scan the world for information that is relevant to them (Reed, 1996, pp 18–19). For the creative practitioner, the world is an endless supply of resources for creativity. In her autobiography, choreographer Twyla Tharp describes how she perceives her environment in terms of the affordances it provides to think in new ways about her choreography.

Everything that happens in my day is transactional between the external world and my internal world. Everything is raw material. Everything is relevant. Everything is usable. Everything feeds my creativity. But without proper preparation, I cannot see it, retain it, use it (Tharp and Reiter, 2003, p. 10).

By “proper preparation,” she likely refers to the necessary skills and expertise required for creativity within her domain. However, preparation also describes her mindset; the environmental scanning she conducts is goal-directed, focused by interest and concern for dance.

Anecdotes describe how people develop a breakthrough idea through what seems sheer good luck. These stories feed myths of creativity as divine inspiration: Archimedes in the bath as he solves a method for measuring the volume of irregular objects; Newton’s observation of a falling apple as inspiration for his universal theory of gravity (Epstein, 1979), and Flemings discovery of penicillin in a moldy petri dish (Bennett and Chung, 2001, p. 168), to name a few. Feynman recounts the fortunate day he was in a cafeteria when someone threw a plate in the air; he credits this serendipitous event as inciting a process that led to the Nobel Prize (Feynman and Leighton, 1997, pp 171–174).

…So I got this new attitude… I’m going to play with physics … Within a week I was in the cafeteria and some guy, fooling around, throws a plate in the air. As the plate went up in the air I saw it wobble, and I noticed the red medallion of Cornell on the plate going around. It was pretty obvious to me that the medallion went around faster than the wobbling…I had nothing to do, so I start figuring out the motion of the rotating plate. (Feynman and Leighton).

Feynman reached a point of frustration in his research program and decided to deal with his inability to make scientific progress by looking for opportunities to “play with physics.” In solving the spin to wobble ratio of the plate he developed a complex equation, which led to calculation of electron orbits and breakthrough in his research program. Anecdotes like Feynman’s may capture our imagination because, at first glance, they seem like the happy accident of good fortune. But, as Louis Pasteur is often quoted, “*Dans les champs de l’observation le hasard ne favorise que les esprits préparés*.” (Where observation is concerned, chance favors only the prepared mind.) Feynman was seeking affordances to help him play with physics, shaped by a general concern for his research program. Creative people may talk about being lucky, but luck, it has been said, “is the residue of design.”^10^ They become experts at perceiving the opportunities afforded by their resource-rich environments as they move within and between them.

**Theme 4: People actively scan their environments, and seek out new environments, for opportunities to perceive problems or ideas in new ways.**

### Settings Shape Perceiving-in-Action: Creativity as Enactive Cognition

The foundational principle behind enactive cognition is that perception and cognition depend upon a person’s interactions with the world (Varela et al., 1991). People create their own experiences through their actions; perceptions are shaped by what they do, how they do it, and what they anticipate doing (Noë, 2004). Personal accounts describing embodied and embedded experiences during creativity also fit the enactive paradigm. Evidence of embodied cognition was found in narratives about intuitive processes during stages categorized as *generating modes* of thinking when people *think-in-action* through activities like writing, drawing, or model making. Embedded cognition evidence was more typically found in stories of people explicitly using epistemic actions during *elaborating modes* of creativity *when they change the context of a situation to perceive new affordances* within a setting — such as through abstraction with tools and materials or analogy using artifacts — or by changing settings. The third mode of creative ideation, incubating, is generally understood to involve sub-conscious (or semi-conscious) mental processes, however, evidences suggests it is sensitive to environmental conditions (Dijksterhuis and Meurs, 2006; Sio and Ormerod, 2009; Leung et al., 2012). In this section, the enactive perspective is discussed in terms of how it may help shed light on relationships between different modes of creative ideation and the environmental conditions supporting them.

#### Role of Physical Conditions in Complementary Processes of Generating and Elaborating

People engage in complementary intuitive and explicit processes when working on a creative problem; breakthrough emerges over time, with incremental insights constituted by engagement with tools, materials, and features of the architectural environment. During flow, creative practitioners often describe feeling part of the product of their ideation (such as Berger’s drawing), however, during elaboration modes their relationship to creative work changes; it becomes an object of explicit and critical evaluation. Aleksakova, an architect, describes how through intuitive and dynamic process of perceiving and acting, she notices an unexpected outcome of cutting, altering her relationship with the product of creation; she no longer feels a part of it. The moment of surprise triggers a process change from intuitive generating to explicit elaborating.

*You stop thinking*,

*You just look at the piece of foam and you try to make it beautiful*,

You cut.

*Sometimes you slice something*,

*And then another thing*,

And ou-u-u-p-p-p something is there.

And you think:

‘Oh, that’s interesting;’ it’s there. (Yaneva, 2009, p. 57)

During flow (which she explains happens when “you stop thinking”), knife and foam are transparent equipment allowing Aleksakova to externalize thinking about a creative problem; she describes actions and perceptions merging as an idea takes shape from the process. Each action, guided by intuitive response to a previous action, is in pursuit of the goal to “make it beautiful.” When goal-directed *expectations of an action* do not match *perceived result* of that action (“something is there”) flow processes break down and surprise triggers explicit processes of elaboration (“you think: ‘oh, that’s interesting’…”). *The enactive perspective reveals how physical conditions in a creative situation can curtail one mode of creativity and trigger another.*

Although people can choose to stop intuitively working on a problem, and decide to critically consider the outcome of their work, first person accounts of creativity reveal how movement between modes of creativity is often *not a conscious decision*. This perspective is not evident in the creative stage models, yet it has relevance for design strategies intended to improve creative productivity. Complementary relationship between modes of intuitive immersion and explicit elaboration suggests that typical sequencing of creative stages (generating, incubating, and elaborating) may not reflect the iterative ways people transition between them. It also helps highlight differences in environmental conditions supporting each of the modes — and implications this might have for workplace designs.

**Theme 5: People’s perceptions of affordances in their environments depend (in part) on their activities and mode of creative thinking.**

Integration of the embodied, embedded, and enactive perspectives with respect to creative cognition reveals overlaps and disparities between person–environment relationships among different modes of ideation (see **Table 2**). Essential to the intuitive immersion of creative flow is tools and materials functioning as transparent equipment. Tools and materials may also be embodied during explicit elaboration, however, this is not critical, as it seems to be for flow. Failure of a tool (i.e., when a nib breaks) or unexpected outcome of working with a material (i.e., when cutting foam transforms the material in unanticipated ways) will often *engender* the elaboration mode. During elaboration modes tools and materials may be critically regarded as things to think with. (For example, a musician may pick up an unfamiliar instrument to explore an idea for a composition.) Settings for intuitive flow must protect the creative practitioner from interruption or distraction^11^ and support the focused attention required through familiar and comfortable tools, furnishings, and environs. These conditions are not critical for elaboration, which instead benefits from unfamiliar environments and resources, helping the creative practitioner perceive an idea or product in new ways. How people perceive their environment is, in part, determined by their mode of creative thinking and, in turn, their mode of thinking may be influenced by conditions in their physical environment.

**Table 2:** The 3E’s and modes of creative ideation: summary of themes.

3E Theories	Generating	Elaborating	Incubating
	Intuitive immersion in creative flow	Explicit evaluation and exploration of an idea	Semi-conscious rumination about an idea

**Embodied**	**Theme 1:** Tools and materials are ‘transparent equipment’ when people *see through them* to the task at hand, extending sense of the body.	Tools and materials may be embodied, but this is not critical to the process.	Tools and materials are likely embodied when working on mundane tasks unrelated to the creative problem
	**Theme 2:** People incorporate features and sensory attributes of settings into ritualistic behaviors to psychologically prepare for creative efforts, integrating them into concept of creative self over time.		People describe rituals (like walking or riding a train) and favorite settings with similar sensory qualities to help them incubate; they do not, however, express integration of these places into concept of creative self.

**Embedded**	*Seeing with* materials sustains complementary processes of acting and perceiving through continuous reciprocal causation.	**Theme 3:** People *see with* tools and materials and *see as* objects and features of their environment to understand complex problems and creative situations in new ways.	
		**Theme 4:** People actively scan their environments, and seek out new environments, for opportunities to perceive different affordances in problems or ideas.	Environmental cues positively influence insight during incubation.

**Enactive**	**Theme 5:** People’s perceptions of affordances in their environments depend (in part) on their activities and mode of creative thinking.		
	**Theme 6:** People change conditions in their environments, or move to new environments, to help them transition between creative modes of ideation.		


#### Autopoiesis, Niche Construction, and Creative Ideation

Central to the enactive thesis is a systems approach to understanding human cognition. As developed by Varela et al. (1991) its core concepts are influenced by *autopoiesis* (Varela et al., 1974), considering living organisms as “autonomous systems” who “regulate their interactions with the world in such a way that they transform the world into a place of salience, meaning, and value” (Thompson and Stapleton, 2009, p. 25). By “transforming the world” people create their own “milieu” (i.e., cognitive niche). Cognition, from the enactive perspective, is structural coupling between brain, body, and world; *“it is the relational process of sense-making that takes place between the system and its environment”* (Thompson and Stapleton, 2009, p. 26). Stories reviewed in this paper, organized by common mode of creative thinking and theories of physically situated cognition, begin to reveal how creative practitioners exploit, transform, and move between settings, constructing cognitive niches to engender, sustain, and enhance different modes of creativity. Creative practitioners describe choosing similar types of places where they feel most creative and *vote with their feet* when creativity wanes by seeking out new places to work.

People quickly identify places that are not conducive to creativity and will change settings to keep creative productivity high (Buttimer, 1983). Kipling (1937) describes how his creative “Daemon would not function in brickyards” so he “walked the other way.”

I wrote a tale … in a brickyard…. It turned out a painstaken and meritorious piece of work, overloaded with verified references, with about as much feeling to it as a walking-stick…. Evidently my Daemon would not function in brickyards or schoolrooms. Therefore, like Alice in Wonderland, I turned my back on the whole thing and walked the other way.

People periodically change environments (by moving to a new place or by reconfiguring an existing space), leading Buttimer (1983, p. 59) to suggest “creative work demands quiet and privacy, but also needs movement and a sense of change…”. The latter seems to particularly be the case when people have reached a point of frustration on a creative problem. The famous saying that creativity happens in the bed, bus, and bath (Dart, 1989) was inspired by some of the most compelling stories of creativity describing a moment of insight — coming (seemingly) from out of the blue (such as Nikola Tesla’s idea for alternating current, which came to him during a walk), during a dream-like state (like Kekulé’s insight into the ring-like structure of benzene while dozing in front of the fire), or when engaged in an unrelated activity (such as the famous myth of Archimedes’s “eureka” moment during a bath). Wallas (1926) coined the term incubation to describe this stage of creativity. Although the incubative process is not well understood, it is believed an instrumental part of ideation and thus warrants discussion here.

Incubation occurs when conscious work on a problem ceases, particularly during period of indecision (Cohen and Ferrari, 2010). Studies find insight improved when people work on unrelated mundane (low-cognitive load) tasks during incubation (Dijksterhuis and Meurs, 2006; Sio and Ormerod, 2009) or when environmental cues are encountered immediately before or during incubation (Sio and Ormerod, 2009). Incubation may involve embodied tools or materials when a creative practitioner uses them to engage in unrelated work, but this does not seem a necessary condition. It may be a form of embedded cognition when people have creative breakthroughs in response to environmental cues. Stories where people incorporated cues from their setting to yield insight (such as Farnsworth’s) suggest elaboration and incubation may be related modes of critical reflection during creativity – one involving explicit cognitive processes and the other sub-conscious (or semi-conscious) reflection on a creative problem or idea. A striking theme among personal and biographical accounts of creativity is similarity of settings and activities where people experienced creative insight during incubation.

Incubation stories overwhelmingly describe insight happening while walking or riding a bus, carriage or train. Von Helmholtz claimed incubation did not occur when he was tired or while at his worktable, but walking outside encouraged it (Wallas, 1926, p. 80). Poincaré (1954, p. 26) also described insight occurring during incubation when he took a break from work and went for a walk, rode the bus, or when involved in unrelated activities while serving in the military. The train is identified as a productive workplace in both Buttimer’s (1983) report on 45 creatives from diverse disciplines and in Törnqvist’s (2004) analysis of biographies written about Nobel Laureates. They are so often referenced in personal accounts that Harding and Nichols (1948) suggest the rhythm of transportation modes may induce in creative practitioners a hypnotic state conducive to ideation. Whether motion, background noise [as suggested by Mehta et al.’s (2012) study mentioned previously], or other environmental qualities, people seek out similar sense-giving spaces to invite incubation. Thus incubation may be affected by environmental conditions under which it takes place, however, these examples do not obviously fit the enactive paradigm described by Varela et al. (1991).

Anecdotes suggest settings may play a role in encouraging or sustaining incubation; given limited knowledge of the mechanisms behind the intuitive process, there is not enough evidence to determine if it may be a form of enactive cognition. For alternative explanation, Clark (1999) argues complex “representation-hungry” problems requiring abstraction or imagination may involve “off-line reasoning” (in other words, mental representation, which is antithetical to the enactive thesis). Ward et al. (2011, p. 375) suggests it is not “bodily activity itself but our practical knowledge (which need not be verbalized or in any way explicit) of our own possibilities for action” that constitutes understanding. In contrast, Bergen’s (2012) embodied simulation hypothesis proposes people do not rely on mental representations during off-line thinking, rather they imagine virtual experiences; abstract thinking may be grounded in action through metaphor (Lakoff and Johnson, 1980). Even if incubation does involve “off-line reasoning,” stories of creativity suggest certain environmental conditions might inhibit the process (such as by demanding too much attention) or provide qualities that people find support their ability to incubate (such as spatial configurations that invite walking)^12^.

**Theme 6: People change conditions in their environments, or move to new environments, to help them transition between creative modes of ideation.**

## Toward an Ecological Model of Creativity

Analysis of first person and biographical accounts reveals several things of potential relevance to the design of settings to support creativity. First, there is evidence that creative processes are embodied and embedded in, and enacted by architectural settings. This suggests *architectural designs have the potential to positively or negatively impact user creativity*. Second, themes from 3E analysis, organized with respect to the creative modes in **Table 2**, illustrate how *a single mode of creativity may involve multiple forms of physically situated cognition* (e.g., the elaboration mode may be embodied, embedded, and enactive). Although it is useful from the perspective of analysis to separate the 3E’s, in reality they are often integrated during *creativity in the world.* Development of a theoretical framework to guide design strategies must account for this. Third, *how people perceive features and attributes of their environments is shaped by their mode of creative thinking*. Thus how and why people use settings must be examined with respect to each mode of creativity. Fourth, *people change their environments to help them transition between modes of creativity*. The analysis in the paper reveals how creative modes may be supported by different environmental conditions. This suggests the environments which support one mode of creativity may inhibit another. For example, workplaces designed to maximize impromptu social interaction may be effective for elaborating modes, but at the cost of inhibiting (or at least harming) generating modes. Finally, it should be noted that it is beyond the scope of this paper to theorize whether *all* creative process are physically situated or if some processes (such as incubation) are *always* physically situated, however, evidence gathered thus far suggests dynamic relationships between person and environment are instrumental for creative practitioners during modes of creative ideation.

### Linking Process and Place

The aim of analyzing creative processes through the lens of the 3E’s was to look for evidence of physical situatedness as a first step toward developing a theoretical framework useful for informing architectural design strategies. The remainder of this section describes how findings from the analysis are used toward this goal. First themes from analysis inform three propositions about person–environment relationships during creativity as follows:

#### Proposition 1

Creative cognition is *embodied* when people *see through* tools and materials while intuitively perceiving-in-action, deepening immersion in the creative process and extending sense of the body during creativity.

#### Proposition 2

Creative cognition is *embedded* when people *see with* or *see as* tools, materials, decorative objects, or other features of their settings as *things to think with*, thereby extending their capabilities to understand a complex problem.

#### Proposition 3

Creative cognition is *enacted* when people construct *cognitive niches for creativity* by interacting with, altering, and moving between settings to engender, sustain, and enhance different modes of creative thinking.

**FIGURE 3:**
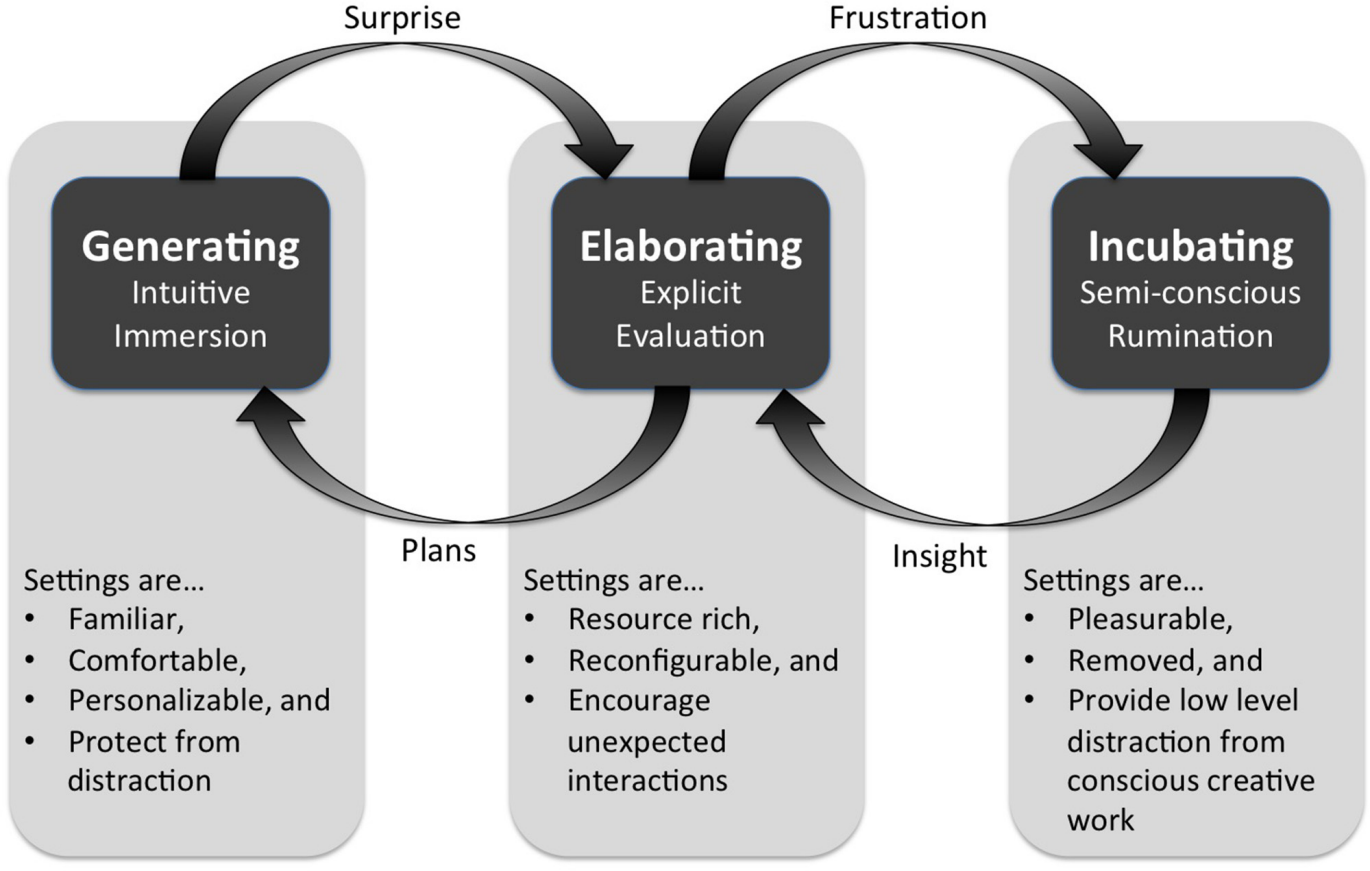
**Linking creative process and place**.

Second, modes of creative ideation are linked with environmental conditions (**Figure 3**). As mentioned in the beginning of this paper, (a) there has been little integration between creative process and press research streams, (b) press research has focused primarily on the social context of creativity, and (c) stage models describe purely mental processes with little incorporation of physically situated theories of cognition. Existing stage models of creativity are not useful for informing architectural design strategies because they neither adequately identify and describe physical activities involved in the sub-processes of creativity nor sufficiently explain the relationships between creative stages or how people move between them. **Figure 3** illustrates through a conceptual diagram those process–place relationships described by creative practitioners as they engaged in modes of creative ideation. The diagram highlights how each mode is supported by different environmental conditions. (A few key setting qualities are provided to illustrate this point.) It also describes relationships between ideation modes. Although people may consciously choose to move between modes, analysis reveals how perceived outcomes of physically situated cognitive processes can curtail or engender modes. This diagram illustrates what creative practitioners have described in these situations. For example, the generating mode of intuitive immersion is curtailed by an unexpected outcome (e.g., Aleksakova’s surprise after cutting the foam) and this triggers the elaborating mode to explicitly explore and evaluate the surprising situation. If the creative practitioner is unable to garner new insight into the situation through exploration and evaluation, frustration may curtail the elaborating mode and trigger incubation (e.g., as Feynman described deciding to walk away from his research and ‘play’ with physics). During incubation the creative practitioner continues to work sub-consciously or semi-consciously on the problem until moment of insight. Insight engenders the elaborating mode to determine its merit and, if suitable, is used to inform a new plan or goal from which to initiate the generating mode. Poincaré (1954) describes this iterative process of moving between modes of generating, evaluating, and incubating as he worked to solve a mathematical problem.

**FIGURE 4:**
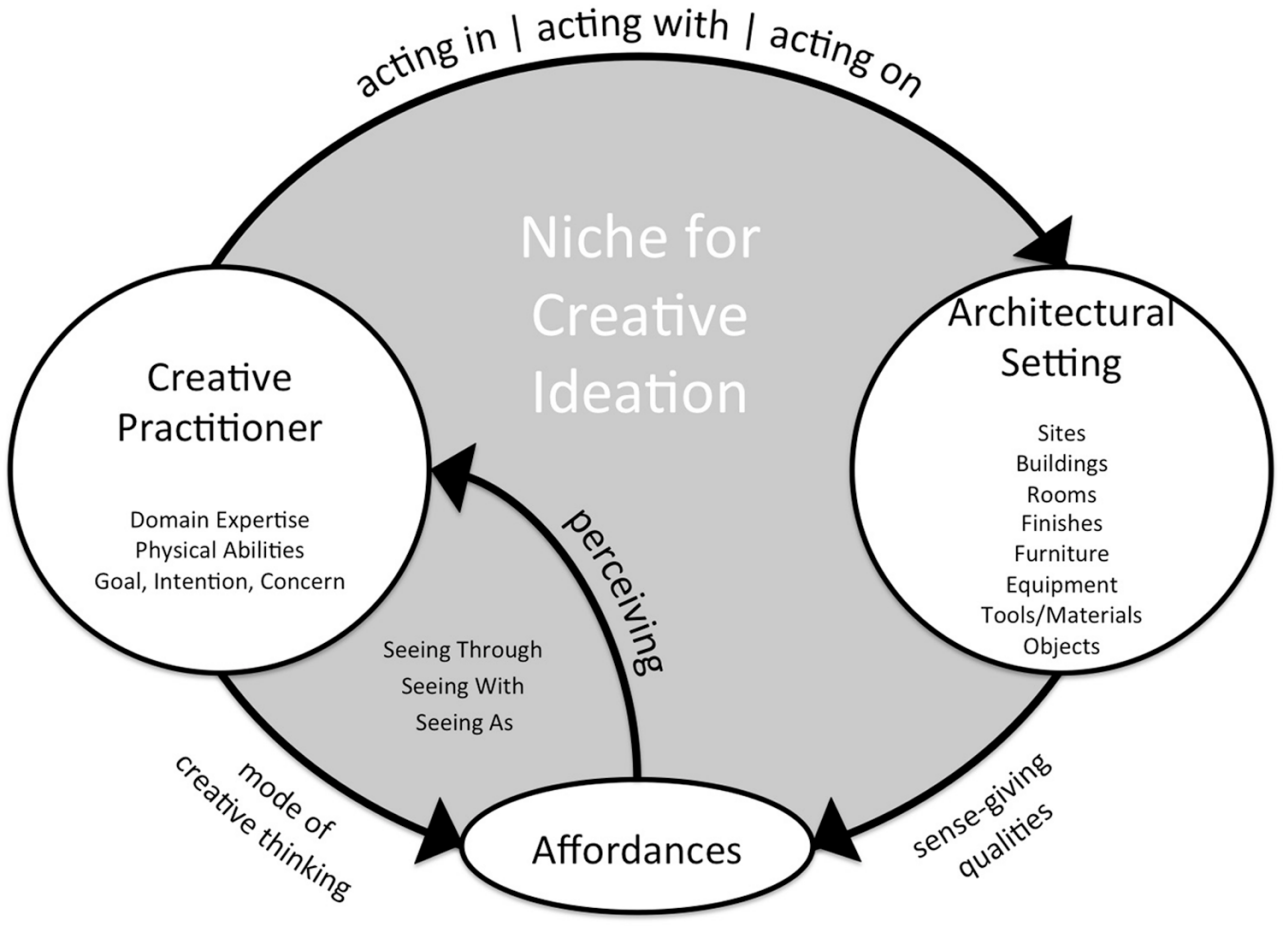
**Framework for an ecological model of creativity**.

Finally, drawing on compatibilities between enactive cognition and Gibson’s affordance theory from ecological psychology, and informed by the three propositions and process–place diagram (**Figure 3**), a preliminary framework for a dynamical systems model (**Figure 4**) illustrates person–environment interactions during creative ideation. Central to an ecological model of creativity is the transactional relationship between person and setting during creativity; people construct cognitive niches for creative modes through their actions within spaces, with artifacts and features of their setting, and by moving from one space to another. This framework intends to provide a starting point for organizing existing research and informing new studies to better understand the relationships between architectural design strategies and user creativity toward developing an ecological model of creativity.

Niche construction is a concept borrowed from evolutionary biology and ecology describing how organisms change environmental conditions to increase their chances of survival (O’Brien and Laland, 2012); they adapt to problematic conditions in their environment through modifications they make to it. In cognitive science the concept is commonly used to describe how people off-load mental work to their environments (e.g., through epistemic actions, externalizing ideas through model making or diagramming, etc.) to extend cognitive capabilities (Clark, 2008a). The framework presented here merges the ecological and cognitive perspectives to better understand (1) how architectural designs impact users’ creative processes and (2) how users exploit, alter, and move between settings to increase creative productivity. It begins to define variables involved in creative niche construction: (1) factors in the architectural setting and their sense-giving qualities, (2) characteristics of the creative person and his/her mode of creative ideation, (3) affordances (i.e., opportunities for action) offered by qualities of the architectural setting with respect to the creative person and mode of ideation, and (4) actions of the creative person that change the architectural setting, thereby impacting affordances offered.

#### Architectural setting

An architectural design is an example of niche construction in the biological sense; a building protects inhabitants from extreme weather and other safety risks, provides comfort through furnishings, equipment to prepare and serve food, a place to bathe, etc. It is a milieu, exerting environmental pressures on users through spatial configurations and sense-giving qualities. Areas of concern for architectural design professionals define the different variables of the architectural setting in this framework. For example, designs to support the generating mode could be considered from the perspective of site (e.g., the site for the Salk Institute is in a low density, quiet area, and on a cliff overlooking the ocean), buildings, (e.g., Kahn oriented buildings to maximize views toward the ocean), rooms (e.g., he angled scientists offices to provide a window in each, framing the inspirational view), and so forth. People also exert pressures on the architectural settings they use (Brand, 1994); building and user engage in an ongoing reciprocal relationship. For example, Kahn expected users would reconfigure the laboratory spaces at the Salk Institute and designed them to facilitate flexible spatial configurations. However, users have constructed private offices in these buildings, a pressure on the space he did not anticipate. This framework could be used to examine user rationale for the changes they make in and to their settings and how these changes, in turn, impact creative processes. Architectural settings inspire and constrain behavior and cognition through the affordances they offer, and users, by actualizing affordances, change conditions in their environments thereby shaping affordances available to them.

#### Affordances

This framework describes affordances as relationships between sense-giving qualities of an architectural setting with respect to the personal skills and abilities of its user, providing opportunities for creative thinking-in-action. Affordances may invite behaviors (e.g., through spatial configurations encouraging social interaction) or aesthetic appreciation (e.g., through forms and materials). During creativity, room finishes or features afford protection from unwanted distraction (such as Proust’s cork-lined walls) or inspirational views (like Kant’s of the church tower). Decorative objects (for example, those on Kipling’s desk) afford rituals initiating creative efforts, and familiar tools afford perceiving-in-action during flow. Affordances exist whether or not they are perceived or actualized (used). For example, a twig dipped in ink affords writing, even if not perceived as such. Conversely, a pen with a broken nib does not afford writing.

#### Creative practitioner

Creative people have unique expertise^13^ (including domain knowledge and personal experiences) and psychomotor abilities, which, in part, determine affordances offered by their environments and how these are perceived and actualized. For example, a musician perceives a conch shell affords playing and he actualizes that affordance when he *sees through* it in the process of composing music. A writer perceives the shell as something she collected as a child during walks with her mother and actualizes the affordance when she *sees with* it, evoking memories she documents in her story. An architect perceives the shell as an enclosure and actualizes the affordance when he *sees as* it to design the form of a new building. *Seeing*, used figuratively in the model, refers to all ways of perceiving (not exclusively visual), consciously and subconsciously, through the sensorimotor system. Affordances depend upon a person’s goal or intention toward, or concern about, a creative situation, framed by the mode of creative thinking.

#### Actions

Finally, the model describes how people make sense of complex and ill-defined problems through their actions, which alter affordances perceived and actualized in the situation. People have autonomy (Thompson and Stapleton, 2009), or agency (Gibson, 1977; Reed, 1996), to seek out environments (like trains) that help them be creative, develop behaviors to fit their environment (such as ritualistic cleaning of work surfaces, setting out favorite tools, gazing at an inspirational view) and alter environments to suit their needs (to address ‘poor fit’ such as when Kant had his neighbor’s tree cut down after it blocked his tower view, or through personalization to inspire creativity such as the meaningful objects on Kipling’s desk.)

#### An example

Feynman’s story of the spinning plate illustrates how the framework might be used to guide understanding and empirical examination of physically situated processes involved in creativity. He, as a creative practitioner, has domain expertise in physics. In a period of frustration, he stops working on his research problem and decides to ‘play’ with physics. He walks around the Cornell University campus (changing his environment by moving to a new setting) with an intention to play with physics. When he arrives in the cafeteria he perceives a plate tossed into the air by a student. Because of his domain expertise, he notes the rate at which it spins and wobbles are different. He perceives this as an opportunity to play with physics by figuring out the ratio. Significantly, he perceives the plate’s affordance *while he is in incubating mode*, which was engendered when he stopped working on his research. Although the plate might seem a new creative problem to pursue, it is a process through which he gains insight on his research program. He *sees with* the plate to develop a new perspective on his creative problem. Working out the ratio, he devises a plan allowing him to resume productive work on his research, eventually leading to a breakthrough and the Nobel Prize.

Stage models of creativity do not capture the iterative, physically situated nature of incremental breakthroughs on a creative problem that creative practitioners describe. Stories like Feynman’s, Poincaré’s, and others help to reveal the dynamical relationship between person and environment during creativity. Gibson’s affordance theory of visual perception provides a foundation upon which to develop understanding of this relationship. However, to empirically examine the creative process as a form of physically situated cognition, it must be extended to include key personal characteristics and environmental factors impacting creative processes and outcomes. The framework proposed in this paper aims to provide a first step toward that goal. By linking process, and place, it may provide a useful structure to bridge research in creativity, cognitive science, and architectural design toward developing an ecological model of creative processes.

## Limitations and Future Research

Analysis presented in this paper was limited to examination of existing first-person and biographical accounts of creativity by creative practitioners and therefore excluded perspectives from creative practitioners who were not compelled to write about their processes. It is possible those who write about creativity may not adequately represent the entire creative practitioner population. Most narratives were written by people who had quite a bit of freedom to work where, when, and how they wished. This provided a wealth of data, but does not represent the typical corporate office employee.^14^ There are more personal accounts by authors, than, for example, musicians. Authors may be more inclined to write personal accounts or some creative practitioners may feel they have less to write about; there may be people who believe their creative processes are not dependent upon physical conditions and thus are not compelled to write about them. Although efforts were made to include a diversity of creative perspectives (e.g., from the arts, design, humanities, math, and science) not every creative field is represented in this analysis. This paper also aimed to identify domain general (independent)^15^ modes of creativity, thus disciplinary and individual process differences are not considered. Finally, it focused solely on narratives about ideation modes of the creative process; it did not consider other stages of creativity that might also be physically situated (such as problem finding or implementation).

Much more research is needed (and from multidisciplinary perspectives) to better understand how physical contexts impact — and ways architectural designs might support — human creativity. As a small step toward a rather lofty goal, this paper attempts to provide some evidence of how creativity is physically situated in architectural settings. It does so by (1) identifying common (domain-general) modes of creative thinking, (2) organizing first person and biographical accounts describing the things creative people do when engaged in these creative modes, (3) analyzing these through the lens of situated cognition with the embodied, embedded and enactive cognition theses, and (4) illustrating person-environment relationships they describe in a theoretical framework integrating complementary concepts from enactive cognition and ecological psychology. Next steps in this research program include:

(1) *Extending the framework to include problem-finding and implementing modes*, through:
  (a) Analysis of first-person and biographical accounts, to identify other physically situated processes.
  (b) Testing explanatory power against documented impacts of architectural designs on creative productivity.
  (c) Testing predictive power through case and quasi-experimental studies of creative practitioners in workplace settings (pre- and post-occupancy).
(2) *Identifying additional environmental mechanisms* relevant to understanding potential impacts of architectural design strategies including:
  (a) Other environmental factors involved in engendering, sustaining, and/or inhibiting modes of creativity and relationships between them.
  (b) Design variables in architectural settings with respect to modes of creativity.
  (c) Organizational factors impacting how people use settings.
(3) Develop a dynamic systems model integrating person, cognitive, social and physical factors
  (a) Including separating domain-general, domain-specific, and subject/personal processes involved in physically situated processes.

## Conclusion

Anecdotes about creativity suggest people’s processes involve embodied, embedded, and enactive forms of cognition, with the intertwined nature of thinking and acting a common theme. The physical context of creativity, including architectural settings where people work, remains largely unexamined — in part because of the complexity involved in empirically studying it. Economic pressure on companies to capitalize not only on employee creative productivity by also on every square foot of floor space reveals the untapped potential of architectural designs to add creative value to organizations (De Paoli et al., 2013). Yet there is no theoretical framework appropriate for guiding design decisions or predicting post-occupancy impacts in spaces to support creativity. Roughly 30 years ago, Gibson (1976, p. 413) complained “architecture and design do not have a satisfactory theoretical basis” and many in the profession feel this statement holds true today (Hensel et al., 2009; Lang and Moleski, 2010). Complementary concepts from Gibson’s theory of affordances and the enactive thesis of human cognition may together begin to provide the framework for a functional theory linking cognition, behavior, and environmental design. The model proposed in this paper suggests the benefits such an integrative approach could have for architects and creativity researchers in guiding future scientific research and design practices.

## Conflict of Interest Statement

The author declares that the research was conducted in the absence of any commercial or financial relationships that could be construed as a potential conflict of interest.

## Footnotes

1 Creativity is typically described as *extraordinary* (defined by ideas that are a significant departure from those in the field) or *everyday* (encompassing ideas that are original to the person or persons who conceived them (Csikszentmihalyi, 1996). Places designed to support creativity generally target users whose work is deemed creative by others, but may not be extraordinary (i.e., creative practitioners).

2 For example, the Domain-Individual-Field-Interaction (DIFI) model (Feldman et al., 1994) or Amabile’s (1996) componential model linking person, process and (social) press.

3 For example, Van Gundy (1987), Mumford et al. (1991), Feldman et al. (1994), and Isaksen et al. (2000) in **Table 1.**

4 Eminently creative people are frequent subjects of empirical investigation, but creativity is understood to involve ordinary cognitive processes (Ward and Kolomyts, 2010).

5 For example, a version of the Wallas model is incorporated in Amabile’s componential model and the DIFI model.

6 A growing number of researchers challenge cognitivist categorical structural (i.e., people create mental models of the world from which they plan and problem-solve) and centralized processing perspective (i.e., first people perceive, then they think, and finally they act; Thagard, 2005).

7 The *extended mind thesis* is often included as a fourth “E,” but not part of this analysis.

8 Norman popularized the term in his book “The Design of Everyday Things” (Norman, 1998). Gibson considered affordances all actionable properties of the physical environment, whereas Norman’s defined them as perceived actionable properties.

9 For other examples, artist interviews (Fig, 2009) or Csikszentmihalyi (1996, p. 119) quoting Barry Commoner describing how his favorite fountain pen allows his ideas to flow, whereas a ballpoint pen does not offer the same experience.

10 Attributed to Branch Rickey, also John Milton.

11 Importance exemplified by Coleridge’s (1816, pp 50–57) famous account of interruption while composing *Kubla Khan*.

12 For example, experimental studies by Leung et al. (2012) suggest spatial configurations and ways people move through them influences quality and quantity of ideas generated.

13 For example, research suggests people’s visual perception changes as they develop expertise in a domain.

14 Because office worker behavior is tightly governed by organizational culture, this population would not have been useful for the purposes of understanding how creative processes might be physically situated.

15 Scientists debate whether creativity is domain-general or domain-specific, with perspectives influenced by research approaches (Silvia et al., 2009); examination of creative products typically involves domain-specific research whereas person and process research is concerned with identifying domain-general traits and stages.
